# Micro- and Nanoplastics and the Oral Cavity: Implications for Oral and Systemic Health, Dental Practice, and the Environment—A Narrative Review

**DOI:** 10.3390/jfb16090332

**Published:** 2025-09-06

**Authors:** Federica Di Spirito, Veronica Folliero, Maria Pia Di Palo, Giuseppina De Benedetto, Leonardo Aulisio, Stefano Martina, Luca Rinaldi, Gianluigi Franci

**Affiliations:** 1 Department of Medicine, Surgery and Dentistry, University of Salerno, Via S. Allende, 84081 Baronissi, Italy; vfolliero@unisa.it (V.F.); giusydb15@gmail.com (G.D.B.); laulisio@unisa.it (L.A.); smartina@unisa.it (S.M.); gfranci@unisa.it (G.F.); 2 Department of Medicine and Health Sciences, University of Molise, 86100 Campobasso, Italy; luca.rinaldi@unimol.it

**Keywords:** microplastics, nanoplastics, oral cavity, dental materials, dental sources, oral care products, environmental exposure

## Abstract

Background: Micro- and nanoplastics (MNPs) have emerged as increasing environmental and public health concerns. Dentistry contributes to this exposure through polymer-based materials and personal oral care products. This review summarizes the current evidence on the sources, release mechanisms, physicochemical properties, and toxicological and biological effects of MNPs derived from dental sources and oral care products, as well as the synergistic effects of MNP oral exposure with environmental exposure. Methods: An electronic search was performed across the PubMed/MEDLINE, Scopus, and Web of Science databases to identify studies investigating the source, release mechanisms, physico/chemical properties, and toxicological/biological impact of MNPs related to dental materials, oral care products, and the synergic effects of MNPs oral and environmental exposure. Results: MNPs are released in the dental setting from resin-based composites, clear aligners, and prosthetic and impression materials through degradation, wear, and handling processes. Home-use products like toothpastes, toothbrushes, floss, and mouthwashes contribute to chronic oral exposure. Evidence from in vitro, in vivo, and human biomonitoring studies supports the biological activity and systemic distribution of MNPs. Despite this, clinical awareness remains limited, and regulatory oversight insufficient. Conclusions: Dentistry is both a source and vector of MNP exposure. Encouraging the use of safer, MNP-free materials, and raising awareness among dental professionals, may support more responsible and health-conscious practices. Further research and alignment with global policy strategies could help guide future innovation and risk mitigation in the dental field.

## 1. Introduction

Plastics contain over 13,000 chemicals, of which at least 3200 are known or suspected to be hazardous to human health or the environment, including carcinogens, neurotoxicants, endocrine-disrupting chemicals (EDCs), and persistent organic pollutants (POPs) [1].

Microplastics (MPs, <5 mm) and nanoplastics (NPs, <1 µm), collectively referred to as micro-nano-plastics (MNPs), are pervasive environmental contaminants that have been detected across all ecosystems and within the human body, including blood, placenta, lung tissue, breastmilk, and stool. MNPs are bioavailable, capable of crossing epithelial barriers, and systemically distributed, with the potential to accumulate in tissues and induce multi-organ toxicity [2,3,4]. Increasing evidence supports the role of MNPs as biologically active pollutants, contradicting the earlier assumption of their inertness. They can act as vectors for environmental contaminants, interfere with cellular homeostasis, and potentially contribute to chronic diseases, including cancer [5,6]. Experimental models have shown that MNP exposure leads to oxidative stress, inflammation, cytotoxicity, and immune dysregulation, as well as disruptions in gut microbiota, impaired fertility, developmental neurotoxicity, and translocation across the blood–brain and placental barriers [7,8,9].

Systemic health risks associated with chronic exposure to carcinogenic chemicals derived from plastics include elevated risks of cancer, respiratory disease, and premature mortality [10,11,12,13].

Oral health risks are linked with the still little investigated oral route of exposure—through ingestion and inhalation of MNPs originating from dental materials and oral-care products—which has been linked to oral mucosal irritation, oral dysbiosis, genotoxic effects on oral cells, and carcinogenic potential [5,14,15,16,17]. Emerging evidence links MNPs to oral carcinogenesis through cytotoxic and genotoxic effects on oral epithelial cells and fibroblasts following chronic exposure to MNPs, which may enter the oral cavity via dental materials, oral hygiene products, and microplastic-containing tools such as polishing pastes and floss [5,8].

Therefore, the present narrative review aimed to summarize the current evidence on source, release mechanisms, physico/chemical properties, and toxicological/biological effects of MNPs derived from dental sources (e.g., composite resins, clear aligners, orthodontic and prosthetic devices, impression materials) and oral care products (e.g., toothpastes, mouth rinses), as well as the synergic effects of MNPs oral exposure with environmental exposure. Particular attention is given to their potential biological impact on both oral and systemic health.

## 2. Materials and Methods

### 2.1. Search Strategy

An electronic search was performed across the PubMed/MEDLINE, Scopus, and Web of Science databases up to 10 July 2025, to identify studies investigating the source, release mechanisms, physico/chemical properties, and toxicological/biological impact of MNPs related to dental materials and oral care products, and the synergic effects of MNPs oral exposure with environmental exposure.

For the electronic search, no restrictions about years of publication were used, and the last update was carried out on 10 July 2025. The following keywords were used to conduct the electronic search, combined with Boolean operators, applying the filter “English language” in all searched databases: (microplastic* OR nanoplastic* OR MNP OR MPs OR NPs OR “microplastic release”) AND (“dental materials” OR “clear aligners” OR “dental wear” OR “oral care products” OR “composite resin” OR dentist OR “dental professional” OR “oral release”).

In addition to the electronic database search, a manual search of the reference lists from the relevant articles was performed to identify further eligible studies. Relevant records were independently screened by three reviewers (F.D.S., V.F., and M.P.D.P.).

### 2.2. Study Selection and Eligibility Criteria

**Figure 1:**
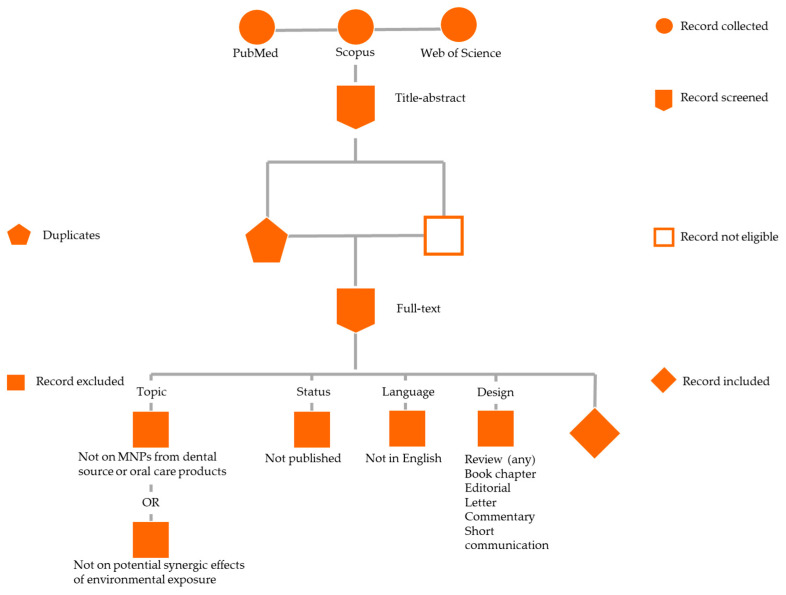
Schematic diagram of the study selection process: phase (I) collection of records from the searched databases; phase (II) records screening by title–abstract: duplicates and records not relevant were eliminated; phase (III) record screening by full-text: records not compliant with the eligible criteria were excluded and records compliant with the eligible criteria were included.

The study selection process was performed by the same three reviewers (F.D.S, V.F., and M.P.D.P) and it is displayed in Figure 1.

Inclusion criteria were as follows: in vitro studies, in vivo studies, or studies involving human participants, including randomized controlled trials, cohort studies, case-control studies, cross-sectional studies, case series, and case reports; studies published in English, addressing the origin, release mechanisms, physico/chemical properties, and toxicological/biological effects of MNPs from dental sources (e.g., composite resins, clear aligners, orthodontic and prosthetic devices, impression materials), oral care products (e.g., toothpastes, mouth rinses), and environmental exposure pathways contributing to oral intake, including studies exploring cellular stress responses, barrier integrity disruption, tissue-level toxicity, and the potential for systemic uptake and bioaccumulation of MNPs. No restrictions were applied concerning publication date, sample size, gender, or age. Therefore, in line with the stated objective of this study, all publications resulting from the aforementioned research were considered relevant, both if focusing exclusively on the release of MNPs from dental sources or oral care products, or if investigating potential synergistic effects arising from combined environmental exposures.

Exclusion criteria were non-English language studies, narrative or systematic reviews, book chapters, editorials, letters to the editor, commentaries, and short communications; studies not already published (e.g., studies accepted or under review); records were also excluded if they did not focus on MNPs originating from dental sources or oral care products, or on mechanisms of release, physico-chemical characterization, or biological/toxicological effects of MNPs, or if they failed to explore potential synergistic effects arising from combined environmental exposures.

### 2.3. Data Extraction, Collection, and Synthesis

Data from the included studies were extracted using a dedicated form developed in Microsoft Excel 2019 (Microsoft Corporation, Redmond, WA, USA) by the same three reviewers (F.D.S, V.F., and M.P.D.P).

All references were organized using Mendeley Reference Manager (version 2.135.0, copyright Elsevier Ltd. (Amsterdam, The Netherlands)).

Given the narrative nature of this review, the data were synthesized qualitatively; a meta-analysis was not conducted due to the early stage of research in this field and the current heterogeneity of study types.

## 3. Results

### 3.1. Human Epidemiological Evidence

Epidemiological studies have begun to shed light on the real-world relevance of MNP exposure in humans, revealing widespread bioaccumulation and potential links to various pathological states, including cancer. Recent investigations utilizing advanced spectroscopic and chromatographic methods have confirmed the presence of MNPs in a broad array of human tissues and biological matrices, such as blood, stool, placenta, lungs, liver, and even cardiovascular and reproductive organs [4,15,18].

One seminal study demonstrated microplastic contamination in human blood, indicating not only oral but also inhalational exposure and systemic dissemination through the circulatory system [4]. This was corroborated by the detection of polymer particles in hepatic tissue obtained from cirrhotic patients, raising concerns about the role of MNPs in hepatic inflammation and oncogenesis [18].

The gastrointestinal tract has emerged as a principal site of MNP accumulation. Clinical studies have identified plastic particles in colonic tissues and feces, with higher levels observed in individuals with inflammatory bowel disease compared to healthy controls [19]. This suggests a potential relationship between chronic inflammation, mucosal barrier disruption, and microplastic retention, all of which are known risk factors for colorectal cancer.

Importantly, MPs have also been detected in the placenta and meconium, confirming prenatal exposure and the capacity for transplacental transfer [20]. These findings imply that fetal development may be vulnerable to MNP-related toxicity, with long-term implications for immune programming and possibly tumorigenesis.

Recent clinical data have confirmed MNP presence in human lung tissues, including specimens collected from individuals undergoing surgery for pulmonary diseases. This suggests that inhaled MPs can penetrate deep into the respiratory tract and persist in the parenchyma, potentially contributing to chronic inflammation, fibrosis, and neoplastic transformation [21,22,23].

The oral cavity is also emerging as a relevant site of exposure. MPs have been identified in human saliva samples, particularly after the use of plastic containers for food storage [24]. These particles may interact locally with mucosal surfaces and contribute to downstream gastrointestinal and systemic exposure. In vitro experiments with gingival fibroblasts exposed to polystyrene NPs showed cytoskeletal disruption, mitochondrial dysfunction, increased reactive oxygen species (ROS) generation, and DNA damage—changes suggestive of chronic inflammatory and genotoxic potential [21].

Animal models of oral exposure have demonstrated deposition of MPs in the oral mucosa, gastrointestinal tissues, and lymphoid organs, along with evidence of epithelial damage and gut barrier impairment. Microbiome analyses revealed dysbiosis characterized by expansion of pro-inflammatory taxa [17]. Observational studies have further shown that individuals frequently using plastic containers exhibit reduced microbial diversity and enrichment of potential pathobionts in their oral and intestinal microbiota [16], reinforcing the hypothesis that habitual MP exposure may contribute to chronic inflammation and systemic disease risk.

Of particular concern are reports documenting MPs within para-tumoral and tumor tissues of prostate cancer patients, suggesting tropism or preferential retention in the tumor microenvironment [14]. Moreover, MPs have been isolated from cardiovascular tissues, including atherosclerotic plaques, raising the possibility of a mechanistic role in chronic vascular inflammation and oncogenic processes [25].

### 3.2. Micro- and Nanoplastics and Human Health: Sources, Routes of Transmission, and Toxicity

Plastics are widely used due to their durability, light weight, low cost, and resistance to degradation [26]. Global plastic production exceeded 348 million tons by 2017 [27]. As plastic waste accumulates, the environment becomes increasingly contaminated with MPs (MPs; 1 µm–5 mm) and NPs (NPs; 1 nm–1 µm), which are now considered ubiquitous pollutants in marine, freshwater, terrestrial, and atmospheric ecosystems [28,29].

#### 3.2.1. Sources

MPs originate from both primary sources, such as cosmetics, exfoliants, and industrial abrasives [30,31], and secondary sources, which result from the fragmentation and degradation of larger plastic debris [29,32]. Secondary MPs predominate in environmental matrices and are considered more harmful due to their persistence and capacity for further degradation [33]. These particles can adsorb and transport hazardous chemicals such as plasticizers, heavy metals, polychlorinated biphenyls (PCBs), and PAHs, thereby facilitating toxic interactions with biological systems [30,34].

#### 3.2.2. Routes of Transmission

Human exposure to MNPs occurs through ingestion, inhalation, and, to a lesser extent, dermal contact [28,30]. Ingestion represents the most prominent route and is supported by the detection of MPs/NPs in drinking water [34], bottled water, seafood [35,36], commercial salts [37,38,39], tea bags and coffee [40], dairy products [41], fruits, vegetables, and honey [38,42,43]. Notably, MPs have been found in human stool [42] and even in placental tissues [20], indicating systemic distribution.

Upon ingestion, smaller MPs (5–20 μm) may cross intestinal barriers via M cells in Peyer’s patches and accumulate in internal organs such as the liver and kidneys [26,44,45]. Chronic ingestion can increase the body burden of plastic-associated chemicals, promote bioaccumulation, and induce tissue inflammation [46,47].

Inhalation of airborne MPs/NPs poses another significant route of exposure, especially via synthetic fibers from textiles, indoor dust, and urban pollution [2,48]. Fibers have been detected in outdoor and indoor air environments, such as Shanghai and Hamburg [49,50], and may also be transported through sea spray [51]. Inhaled particles can deposit in lung alveoli, triggering oxidative stress, inflammation, and possibly crossing the blood–brain barrier [52,53].

Dermal contact, though limited due to the protective stratum corneum, remains plausible especially in the context of cosmetics and personal care products [26,54]. Polyethylene MPs and acrylates used in consumer products can cause irritation or allergic responses [27], and NPs may penetrate damaged or thin skin, interacting with immune cells and generating oxidative stress [27,55].

#### 3.2.3. Toxicity

Following absorption, MNPs can translocate from epithelial surfaces to systemic circulation and accumulate in secondary organs such as the liver, spleen, kidneys, placenta, and brain [4,14,20]. These particles may disrupt immune cell signaling and contribute to the transport of toxic co-contaminants [30,34].

Respiratory toxicity has been observed in both in vitro and in vivo models. Exposure of BEAS-2B epithelial cells to PS-MPs led to oxidative stress and cytokine release [56], and murine studies confirmed particle retention in lungs with autophagic cell death and chronic inflammation [57]. NPs may activate NF-kB and promote proinflammatory responses in alveolar epithelium [58].

Systemically, MPs activate immune responses by inducing IL-6 and TNF-α [59], and may alter T-cell subsets (e.g., Th17, Treg), potentially contributing to immune dysregulation [60].

In the nervous system, MPs have been shown to inhibit acetylcholinesterase in aquatic organisms and rodents [61,62], contributing to neuroinflammation and potential neurodegeneration [3]. In vitro neuronal models revealed oxidative stress and DNA damage following MP exposure [55].

Reproductive and developmental toxicity is increasingly recognized. PS-NPs have been shown to cross the placental barrier and localize in fetal tissues [20]. Human placental models confirm particle retention in the syncytiotrophoblast layer [63], and animal models indicate potential reductions in sperm quality, oocyte integrity, and altered gene expression [64].

Table 1 provides a comprehensive synthesis of the principal exposure and transmission routes for MPs and NPs, identifying key sources, target biological systems, and the associated potential health effects. This format is suitable for inclusion in a scientific manuscript discussing the human health impacts of plastic pollution.

### 3.3. MNPs and Cancer

#### 3.3.1. Accumulation and Bioavailability

The accumulation and bioavailability of MNPs represent a foundational concern in understanding their carcinogenic potential. Upon environmental exposure, MNPs can enter biological systems through various routes, including ingestion, inhalation, and dermal absorption, ultimately leading to their distribution across multiple tissues and organs. Once internalized, their translocation is facilitated by their small size, physicochemical properties, and surface modifications, which allow them to traverse biological membranes and evade immune clearance. Studies have demonstrated that MNPs can accumulate in vital organs such as the liver, kidneys, spleen, gastrointestinal tract, lungs, brain, and even reproductive tissues [66,67].

Orally ingested MPs can undergo gastrointestinal transformation, during which they interact with digestive enzymes, bile salts, and the gut microbiota. These interactions can alter their physicochemical characteristics, potentially enhancing their systemic absorption and toxicological impact [68]. Evidence from in vivo animal models reveals that polystyrene MPs are capable of crossing the intestinal epithelium, entering the bloodstream, and accumulating in secondary organs, with bioavailability being modulated by particle size, surface charge, and chemical additives.

Inhalation is another relevant exposure route, particularly relevant in occupational and urban settings. Airborne MPs have been detected in human lung tissue [22,23], raising concerns about chronic respiratory exposure and subsequent deposition in alveolar regions. Their ability to penetrate pulmonary barriers underscores their potential for systemic dissemination.

In the context of dermal exposure, while intact skin serves as a formidable barrier, disrupted or diseased skin may permit the penetration of smaller NPs, particularly those carrying lipophilic pollutants or solvents. Once within systemic circulation, MNPs may persist due to their resistance to enzymatic degradation, leading to prolonged biological half-life and cumulative toxic burden [69].

Further compounding their bioavailability is the “Trojan horse” effect, whereby MNPs act as vectors for co-adsorbed environmental pollutants—including heavy metals, POPs, and microbial pathogens—thereby enhancing the overall toxicological impact [70,71]. The integration of these pollutants into MNP matrices can increase their lipophilicity, facilitating their absorption across biological membranes and their retention in lipid-rich tissues.

#### 3.3.2. Microbiota Dysbiosis and Cancer

Recent evidence suggests that MNPs disrupt the composition and function of the host microbiota, particularly within the gastrointestinal tract, and that this dysbiosis may contribute to cancer initiation and progression. The gut microbiome plays a key role in modulating immune responses, maintaining epithelial integrity, regulating metabolism, and detoxifying xenobiotics. Perturbations of this finely balanced ecosystem can lead to chronic inflammation, altered immune surveillance, and increased susceptibility to tumorigenesis.

Oral exposure to MNPs has been shown to induce significant shifts in microbial diversity and abundance. Some studies have reported a decline in beneficial commensals, including *Bifidobacterium* and *Lactobacillus*, paralleled by an expansion of opportunistic or pro-inflammatory taxa such as *Escherichia*, *Proteobacteria*, and *Enterobacteriaceae* [72,73,74]. These alterations compromise the gut barrier, promote bacterial translocation, and enhance mucosal inflammation, creating a microenvironment conducive to DNA damage and epithelial transformation.

A compelling mechanistic insight has emerged from studies showing that MNP-induced dysbiosis disrupts specific microbial–metabolite axes implicated in stem cell regulation. For example, the gut microbiota–hypoxanthine–Wnt axis has been shown to regulate hematopoietic stem cell self-renewal, and its disruption by MNPs impairs regenerative capacity and skews hematopoietic lineage commitment—conditions associated with hematologic malignancies [75].

Furthermore, dysbiotic microbiota influenced by MNPs may modulate the bioavailability and enterohepatic circulation of carcinogens and endogenous hormones, thus influencing systemic cancer risk. Changes in microbial enzyme expression, such as β-glucuronidase and azoreductase, can enhance the activation of dietary and environmental procarcinogens within the colon, contributing to genotoxic insult and promoting colorectal cancer pathways [76].

In parallel, microbial-derived metabolites affected by MNP exposure—such as short-chain fatty acids (SCFAs), secondary bile acids, and lipopolysaccharide (LPS)—have been implicated in modulating tumorigenic inflammation and immune suppression. For instance, SCFA depletion and LPS elevation can drive macrophage polarization toward the tumor-promoting M2 phenotype and activate NF-κB signaling, thereby facilitating immune evasion and tumor progression [77,78].

#### 3.3.3. Inflammation and Tumor Microenvironment

The pro-inflammatory properties of MNPs are increasingly recognized as key contributors to cancer development, particularly through their capacity to remodel the tumor microenvironment (TME). Chronic inflammation is a hallmark of cancer and plays a pivotal role in every stage of tumorigenesis, from initiation and promotion to progression and metastasis. MNPs have been shown to trigger both innate and adaptive immune responses, activating inflammatory pathways that, in turn, shape a tumor-permissive niche.

Upon exposure to MNPs, epithelial and immune cells exhibit heightened secretion of pro-inflammatory cytokines, including interleukins (IL-1β, IL-6), tumor necrosis factor-α (TNF-α), and transforming growth factor-β (TGF-β), all of which orchestrate key cellular processes in the TME such as angiogenesis, immune evasion, and epithelial-to-mesenchymal transition (EMT) [79,80]. These cytokines also support the recruitment and polarization of tumor-associated macrophages (TAMs), regulatory T cells (Tregs), and myeloid-derived suppressor cells (MDSCs), collectively fostering an immunosuppressive milieu [79,80]. Increasing evidence has documented MNP-induced accumulation of reactive oxygen species (ROS) [81]. ROS serve as signaling molecules activating redox-sensitive signaling pathways such as MAPK, NF-κB, and HIF1α, which are implicated in inflammation, apoptosis, and carcinogenesis [82].

One pathway implicated in MNP-mediated inflammation is the NLRP3 inflammasome. Activation of this multiprotein complex leads to caspase-1-mediated cleavage of pro-IL-1β and pro-IL-18, resulting in their mature, secreted forms, which amplify local inflammatory cascades. Concurrently, oxidative stress triggers inflammasome activation, such as the NLRP3 complex, which links ROS directly to chronic inflammation and cancer-promoting pathways [77,83].

Experimental models demonstrate that MNPs shed from consumer products activate the ROS/NLRP3/Caspase-1 axis, leading to gut barrier disruption and systemic inflammation [83].

Inflammation driven by MNPs is tightly interlinked with immunoediting within the TME. Persistent exposure to pro-inflammatory mediators promotes genomic instability in resident cells and supports clonal selection of neoplastic populations that evade immune detection [78,84]. Additionally, oxidative stress induced by inflammation creates a positive feedback loop that exacerbates DNA damage and epigenetic alterations.

In terms of immune cell dynamics, MNP-induced inflammation affects macrophage plasticity within tumors. TAMs can be polarized toward a pro-tumoral M2-like phenotype under the influence of IL-10 and TGF-β, which suppress cytotoxic T lymphocyte activity and promote angiogenesis and extracellular matrix remodeling [85,86]. These changes contribute to immune tolerance and enhanced metastatic potential.

Furthermore, MNPs may indirectly support tumor progression by modulating non-immune stromal cells such as cancer-associated fibroblasts (CAFs), which, under inflammatory conditions, can secrete matrix metalloproteinases (MMPs) and vascular endothelial growth factor (VEGF), facilitating tumor invasion and neovascularization [77,87].

#### 3.3.4. Genotoxicity and Epigenetic Alterations

MNPs have demonstrated genotoxic potential across multiple biological systems, raising significant concerns regarding their role in carcinogenesis. Genotoxicity, defined as the capacity to induce damage to the genetic material of cells, can occur directly—through interaction with DNA—or indirectly via the generation of ROS and interference with DNA repair mechanisms. These disruptions may initiate mutagenic events that contribute to malignant transformation, clonal expansion, and tumor heterogeneity.

Upon cellular internalization, typically via endocytosis or passive diffusion, MNPs are trafficked to intracellular compartments, including lysosomes, mitochondria, and the endoplasmic reticulum (ER), where they can trigger structural and functional perturbations. Multiple studies have documented mitochondrial depolarization, increased membrane permeability, and impaired oxidative phosphorylation as consequences of MNP exposure, ultimately resulting in compromised cellular energy metabolism [81].

In vitro and in vivo studies have revealed that exposure to polystyrene and polyethylene-based MNPs leads to chromosomal aberrations, DNA strand breaks, and micronuclei formation in mammalian cells, particularly in hepatic, pulmonary, and gastrointestinal tissues [88,89]. Mechanistically, these effects are mediated by oxidative stress and mitochondrial dysfunction, which compromise genomic integrity through increased base oxidation and impaired repair fidelity [88,89]. Moreover, MNP-induced DNA damage manifests as base modifications, strand breaks, and chromosomal aberrations [88,89]. Disruption of lysosomal integrity and autophagic flux further exacerbates MNP toxicity. Accumulation of MNPs in lysosomes impairs degradation processes and promotes lysosomal membrane permeabilization, releasing cathepsins into the cytoplasm and initiating cell death cascades [88].

Importantly, beyond direct DNA damage, MNPs may interfere with cellular epigenetic regulation, a layer of gene expression control involved in cancer biology. Epigenetic mechanisms such as DNA methylation, histone modification, and non-coding RNA expression can be perturbed by environmental exposures, including those to synthetic polymers. Studies indicate that MNPs alter the methylation status of tumor suppressor genes and oncogenes, modulate histone acetylation patterns, and dysregulate the expression of microRNAs (miRNAs) implicated in cell cycle control, apoptosis, and metastasis [90,91,92].

Further supporting this association, MNPs have been shown to affect the integrity of mitotic spindles and centrosome duplication, thereby increasing the frequency of aneuploidy and chromosomal instability—hallmarks of cancer. The intracellular accumulation of NPs within the nucleus, observed in multiple experimental models, suggests that their small size allows for nuclear translocation, facilitating direct interaction with chromatin and nuclear proteins [93,94]. Additionally, MNPs influence the endoplasmic reticulum stress response by inducing the unfolded protein response [95].

At the transcriptomic level, exposure to MNPs has been associated with deregulation of DNA repair genes, including BRCA1/2, ATM, and MLH1, as well as upregulation of pro-oncogenic transcription factors such as MYC and HIF1α. This molecular signature supports a model in which MNPs promote a “mutator phenotype,” facilitating genetic drift and clonal evolution within precancerous lesions [89,96].

#### 3.3.5. Synergistic Effects with Environmental Contaminants

A growing body of evidence highlights that MNPs not only pose intrinsic toxicological risks but also serve as vectors and modulators for a wide array of environmental contaminants, amplifying their harmful effects. These interactions, often synergistic in nature, occur through adsorption, co-transport, and facilitated cellular uptake of heavy metals, POPs, plastic additives, and EDCs such as phthalates and bisphenols [71,97].

MNPs possess a high surface-area-to-volume ratio and hydrophobic properties, making them ideal carriers for toxic compounds in air, soil, and aquatic environments. Upon ingestion or inhalation, these composite particles bypass physiological barriers and deliver their toxic load directly into cells and tissues. This dual exposure paradigm magnifies cytotoxic, genotoxic, and pro-inflammatory responses, particularly in tissues such as the gastrointestinal tract, liver, and reproductive organs [98,99].

One well-documented example involves the co-exposure of polystyrene MNPs with phthalates, which has been shown to disrupt endocrine signaling pathways crucial to reproductive health. In both zebrafish and rodent models, this combination altered sex hormone levels, impaired gametogenesis, and reduced fertility [100,101]. Additionally, such co-exposures have been implicated in breast and prostate cancer pathogenesis, particularly through the modulation of estrogen and androgen receptor pathways [102].

MNPs also interact with metal ions such as cadmium, lead, and copper, enhancing their cellular uptake and oxidative potential. Studies in aquatic species and mammalian cells demonstrate that such complexes exacerbate redox imbalance, induce DNA damage, and impair mitochondrial function—events central to carcinogenesis [95,103]. The involvement of zinc and copper transporters in these mechanisms has also been observed, linking microplastic-bound metals to disruption of cuproptosis, a recently described programmed cell death pathway relevant in tumor biology [104].

Moreover, the environmental aging of MNPs through UV radiation, oxidation, and mechanical degradation increases their surface reactivity and leaching of chemical additives. UV-aged MPs, for example, have been shown to promote antibiotic resistance gene propagation in microbial communities and facilitate the bioavailability of mutagenic compounds [105]. These interactions underscore the complexity of risk assessment, as MNPs evolve chemically and physically during environmental exposure.

Importantly, these synergistic effects extend to nanomedical concerns. Recent studies suggest that microplastic–contaminant complexes may interfere with the pharmacokinetics of drugs by interacting with human drug transporters or binding serum proteins, thereby altering therapeutic efficacy and toxicity profiles [106,107].

#### 3.3.6. Cancer Promotion and Progression

Beyond initiating carcinogenesis, MNPs may play key roles in promoting tumor progression and exacerbating malignancy through diverse biological mechanisms. Several preclinical and in vivo studies suggest that once internalized, MNPs can contribute to multiple hallmarks of cancer, including sustained proliferation, resistance to cell death, evasion of immune surveillance, and remodeling of the tumTME [93,108].

A key pathway involves the induction of chronic inflammation within the TME. Persistent exposure to polystyrene NPs has been shown to activate signaling cascades such as ROS/NLRP3 and MAPK, resulting in the release of pro-inflammatory cytokines and chemokines that create a permissive niche for tumor cell proliferation and invasion [96,101,109]. These cytokine storms can promote angiogenesis, suppress adaptive immunity, and facilitate EMT—a key step in metastasis.

Recent animal models further substantiate the cancer-promoting potential of MNPs. In murine models of ovarian cancer, chronic exposure to polystyrene nanoparticles accelerated tumor growth, increased angiogenesis, and led to immunosuppressive reprogramming of TAMs, skewing them toward an M2-like phenotype [108]. This immunosuppressive state impairs antigen presentation and facilitates immune evasion by malignant cells.

Additionally, MNPs appear to influence cancer cell plasticity and metastatic competence. For instance, microplastic exposure has been associated with upregulation of mesenchymal markers and increased migratory behavior in cancer cell lines, suggesting enhanced potential for metastatic dissemination [94]. This is supported by evidence from breast and prostate cancer tissues, where MPs have been detected within tumor stroma and vasculature, raising concerns about their role in facilitating tumor spread [5,20].

At the molecular level, MNPs may promote oncogenesis by interfering with transcriptional programs relevant for tumor suppression and cell cycle regulation. For instance, recent omics-based analyses have identified dysregulation of signaling pathways associated with apoptosis, hypoxia, and metabolic reprogramming in MNP-exposed tumor models [67,88].

Furthermore, the interaction of MNPs with cellular membranes and organelles may compromise intracellular transport, lysosomal integrity, and autophagy—mechanisms that are central to tumor cell survival and adaptation to stress. For example, NPs have been shown to impair lysosomal function and inhibit autophagic flux, thereby promoting oncogenic signaling and conferring chemoresistance [5,90].

Although human data remain limited, the extrapolation of findings from in vitro and animal studies strongly suggests that MNP exposure, once established within the host, can act as a promoter of malignancy rather than a passive environmental contaminant. The synergy between MNP-induced inflammation, oxidative stress, immune evasion, and metabolic disruption forms a plausible mechanistic framework for how these particles may contribute to cancer progression and reduced therapeutic efficacy (Table 2).

### 3.4. MNPs and the Oral Cavity: Oral Health Status, Systemic Toxicity, and Cancerogenesis

Cumulative evidence demonstrates that MNPs, especially those originating from oral products, dental materials, and ingestion or inhalation through the oral cavity, pose multiple health hazards [118]. While direct causal links to cancer are not yet fully established in humans, the biological plausibility and accumulation of supportive findings underscore the need for regulation, surveillance, and mechanistic research [118].

Dental-derived MNPs are not confined to local oral environments but possess significant potential for systemic dissemination and toxicity [118]. These findings underscore the need for a comprehensive reassessment of dental material safety, the development of MNP-free alternatives, and long-term epidemiological studies evaluating cumulative systemic risks.

#### 3.4.1. Oral Cancer

Emerging evidence suggests that chronic exposure to MNPs, particularly through the oral cavity, may contribute to the pathogenesis of oral cancer by inducing a spectrum of cytotoxic, genotoxic, and pro-inflammatory changes in oral epithelial cells and fibroblasts. The oral mucosa represents the site of initial contact for ingested and inhaled MNPs, especially considering the high prevalence of these particles in commonly used dental materials, oral hygiene products, and environmental sources such as food packaging and air pollution.

Experimental models utilizing human gingival fibroblasts have demonstrated that polystyrene NPs (PS-NPs, ~100 nm) are capable of penetrating cell membranes and localizing within intracellular compartments, including mitochondria and lysosomes.

Such oxidative damage is particularly relevant in the context of carcinogenesis, as persistent ROS signaling can induce mutations in tumor suppressor genes and activate proto-oncogenes. Additionally, chronic exposure to NPs may compromise the DNA repair capacity of oral epithelial cells, facilitating the accumulation of genomic instability—a hallmark of cancer development.

Further support for this oncogenic potential comes from studies assessing histological and molecular alterations in murine models of chronic oral exposure. Prolonged ingestion of PS MPs (5–10 μm) led to epithelial thinning, goblet cell loss, and disruption of mucosal barriers along the gastrointestinal tract [17,74,119]. While direct evidence of malignant transformation in oral tissues remains preliminary, the observed histopathological changes closely resemble preneoplastic lesions [17,74,119].

In parallel, microplastic retention in human sputum and saliva confirms real-world exposure and suggests a continuous interaction between plastic particles and the oral mucosa [24,120]. Notably, the detection of polyethylene (PE), polyethylene terephthalate (PET), and polystyrene (PS) in human oral secretions—even among individuals without occupational exposure—highlights the ubiquity of these materials in daily life [24,120]. This persistent contact may result in localized inflammation, epithelial dysplasia, and carcinogenic risk over time.

Emerging data also suggest that permeability to several types of nanoparticles may vary across different oral mucosal zones, reflecting differences in epithelial thickness, keratinization, vascularization, and exposure to mechanical and chemical stressors [121]. The keratinized epithelium of the hard palate and gingiva may offer partial resistance to MNPs penetration, whereas non-keratinized regions such as the buccal mucosa, ventral tongue, and floor of the mouth present a thinner epithelial barrier, potentially facilitating deeper particle infiltration and enhanced cytotoxic or genotoxic effects [121]. These anatomical and histological differences should be investigated to determine whether they contribute to site-specific patterns of early dysplastic change in subjects chronically exposed to MNPs.

Finally, the interaction between MNPs and salivary proteins may alter their aggregation state and bioavailability. Proteomic analyses have shown that MNPs can bind to oral mucins and low-molecular-weight salivary proteins, potentially modifying their toxicokinetics and enhancing tissue penetration or cellular uptake [24,122]. These findings raise concerns about the long-term implications of such interactions in the pathophysiology of oral precancer and cancer.

#### 3.4.2. Oral Health

Beyond carcinogenic potential, chronic exposure to MNPs has been associated with a variety of adverse effects on oral health, implicating these ubiquitous particles in the pathogenesis of local tissue dysfunction, barrier disruption, inflammation, and microbiota dysbiosis within the oral cavity.

One of the most significant concerns pertains to the cytotoxicity of MNPs for oral mucosal and stromal cells. In vitro experiments on human gingival fibroblasts exposed to polystyrene NPs (100 nm) have revealed a dose-dependent decrease in cell viability, accompanied by mitochondrial dysfunction and elevated production of ROS [123,124,125]. These effects may compromise the integrity and regenerative potential of the gingival tissue, potentially contributing to periodontal degradation, delayed wound healing, and increased susceptibility to mucosal ulceration or secondary infection.

Salivary studies further support the presence and activity of MNPs in the oral cavity. Recent studies detected common plastic polymers—including polyethylene terephthalate (PET), polyethylene (PE), and polystyrene (PS)—in the saliva of healthy individuals following oral exposure to food-grade plastics [16,126]. The consistent detection of these particles in human saliva confirms the oral cavity as both an entry point and a retention reservoir for MPs.

Crucially, the interactions between MNPs and salivary proteins may potentiate toxicological effects. Proteomic studies have demonstrated that NPs bind to salivary mucins and low-molecular-weight salivary proteins, altering their aggregation state and potentially enhancing mucosal adhesion and penetration [127]. This interaction could disrupt the protective barrier function of saliva, compromise lubrication, and reduce antimicrobial defense—critical for maintaining oral homeostasis.

Furthermore, microplastic exposure may perturb the composition and function of the oral microbiota. While data remain limited, analogous evidence from gut microbiome studies suggests that similar dysbiosis may occur in the oral niche. In particular, prolonged exposure to MPs in murine models leads to a decline in beneficial commensals and an increase in pro-inflammatory microbial taxa, with potential for low-grade chronic inflammation and enhanced risk of periodontal disease [128].

Inflammatory responses triggered by MNPs are likely to further affect gingival and periodontal health. The presence of NPs in oral tissues stimulates the release of pro-inflammatory cytokines such as IL-1β, IL-6, and TNF-α, which are well-known mediators of periodontal tissue degradation. Chronic low-level inflammation in response to persistent exposure could accelerate the progression of gingivitis to periodontitis, exacerbate alveolar bone resorption, and contribute to tooth loss, particularly in vulnerable populations such as the elderly or immunocompromised.

Additionally, experimental studies indicate that NPs can disrupt tight junction proteins and the epithelial barrier, compromising oral mucosal permeability [123]. This phenomenon increases the risk of microbial translocation, immune dysregulation, and exposure to circulating antigens, potentially fostering oral hypersensitivity and autoimmune responses.

#### 3.4.3. Systemic Toxicity from MNPs Derived from the Oral Cavity and Dental Practice/Materials

The systemic toxicity of MNPs originating from the oral cavity—especially those released from dental materials and consumer oral-care products—has emerged as a growing concern within environmental toxicology and translational medicine. While the oral cavity serves as a primary interface for MNP exposure via ingestion and inhalation, evidence increasingly suggests that these particles can translocate across epithelial barriers and exert detrimental effects on multiple organ systems.

Dental materials such as resin-based composites, bonding agents, denture bases, sealants, and orthodontic aligners often contain polymeric compounds that may degrade into micro- or nanoscale particles under the mechanical stress of mastication, temperature fluctuations, or enzymatic action from salivary esterases. Toothbrushing and the use of abrasive whitening products also contribute to mechanical erosion and particle generation, particularly in the sub-10 μm range—particles small enough to penetrate biological barriers [123,129].

Once ingested or inhaled, these MNPs can be absorbed through the gastrointestinal or respiratory epithelium and enter systemic circulation. Experimental data using fluorescently labeled MNPs have demonstrated their accumulation in diverse tissues, including the liver, kidneys, spleen, reproductive organs, and secondary lymphoid tissues, where they may persist due to resistance to enzymatic degradation and evasion of phagocytic clearance [62,76,123].

The systemic toxicological effects of these particles include, but are not limited to, oxidative stress, mitochondrial dysfunction, disruption of the gut–liver axis, immune dysregulation, and endocrine disruption. Studies in murine models chronically exposed to orally administered polystyrene MPs reveal intestinal barrier dysfunction, hepatic inflammation, lipid accumulation, and dyslipidemia—all features of metabolic syndrome with known links to carcinogenesis [59,112].

In the liver, MNP accumulation is associated with increased expression of inflammatory markers such as IL-1β, TNF-α, and TGF-β, as well as oxidative DNA damage and lipid peroxidation. These changes not only compromise hepatic function but also foster a pro-tumorigenic milieu via chronic inflammation and fibrogenesis [18,67].

Additionally, in vitro exposure of human gingival and intestinal epithelial cells to nanoscale polystyrene particles has been shown to disrupt mitochondrial dynamics, reduce membrane potential, increase ROS production, and downregulate genes involved in DNA repair and cell cycle control—mechanisms that collectively prime cells for neoplastic transformation or apoptosis [90,130].

Emerging evidence also implicates oral-derived MNPs in dysregulation of the gut microbiota. Exposure to plastic-associated chemicals alters microbial diversity, reduces populations of anti-inflammatory taxa, and increases the abundance of pro-inflammatory or pathogenic microbes. These changes lead to gut–brain axis disruption, immune imbalance, and low-grade systemic inflammation, with potential implications for autoimmune diseases, metabolic disorders, and even neurodegenerative processes [131,132].

Importantly, the endocrine-disrupting properties of additives commonly found in dental plastics—such as bisphenol A (BPA), phthalates, and benzyl butyl phthalate—further compound systemic risk. These chemicals mimic endogenous hormones and interfere with the hypothalamic–pituitary–gonadal axis, altering thyroid hormone signaling and impacting reproductive health by disrupting gametogenesis and embryonic development [133,134].

Lastly, MNPs may interact with environmental and pharmaceutical agents through the “Trojan horse” effect, whereby the particles act as carriers for heavy metals, POPs, or antibiotics. Such complexes may enhance systemic toxicity, promote antimicrobial resistance, and impair the pharmacokinetics of co-administered drugs [106,135].

#### 3.4.4. Cancerogenesis Potentially Related to MNPs Derived from the Oral Cavity and Dental Practice/Materials

MNPs derived from the oral cavity—particularly those originating from dental materials, procedures, and consumer oral-care products—pose a plausible risk for contributing to carcinogenesis across multiple organ systems. While direct clinical evidence remains nascent, a growing body of in vitro, in vivo, and translational research supports the hypothesis that chronic exposure to these particles through the oral route may serve as an initiating and promoting factor in systemic tumorigenesis.

Dental composites, resin cements, orthodontic appliances, and restorative materials frequently incorporate polymeric compounds such as polymethyl methacrylate (PMMA), polyurethane, polyethylene (PE), polypropylene (PP), and bisphenol A-based monomers. Mechanical degradation, enzymatic erosion, and routine dental procedures (e.g., polishing, scaling, ultrasonic cleaning) facilitate the release of MNPs into the oral cavity, where they are either ingested or inhaled over time [17,129].

Recent investigations using animal models of chronic oral exposure have demonstrated that fluorescently labeled polystyrene MPs (5–10 μm) administered over 60 days accumulate not only in oral mucosa and gut epithelium but also in secondary lymphoid organs and systemic tissues [62,136]. Histological analysis of these tissues revealed epithelial thinning, loss of goblet cells, and compromised intestinal barrier function—hallmarks of a microenvironment conducive to chronic inflammation and pre-neoplastic transformation [62,136].

Furthermore, these studies document significant shifts in gut microbiota, including reduction in beneficial commensal genera and proliferation of pro-inflammatory taxa. This MNP-induced dysbiosis contributes to metabolic derangement, local immune dysregulation, and systemic inflammatory signaling—all of which are implicated in the initiation and promotion of gastrointestinal and extraintestinal cancers [5,62,136].

In human settings, polymeric residues consistent with MNPs have been recovered from the sputum and saliva of individuals using standard oral hygiene products such as whitening toothpastes, mouthwashes, and dental floss, many of which contain abrasive or film-forming plastics [21,129]. Although exposure levels vary, the chronicity and cumulative burden of such exposures raise concern for mucosal retention, translocation to draining lymphatics, and eventual systemic dissemination.

Once translocated beyond the oral cavity, MNPs may exert carcinogenic effects through several mechanisms: oxidative DNA damage, immune modulation, mitochondrial dysfunction, and alteration of gene regulatory networks. In vitro studies on intestinal epithelial cells and hepatocytes show significant upregulation of pro-inflammatory signaling pathways (e.g., NF-κB, IL-6, and TNF-α), suppression of tumor suppressor genes, and increased genomic instability upon MNP exposure [137].

More recently, MPs have been found in para-tumoral and neoplastic tissues in human prostate and colorectal cancers, raising the possibility that MNPs—once introduced via the oral cavity—may not only circulate but preferentially accumulate in tumor-prone sites [14,138]. The potential for MNPs to serve as delivery vectors for co-adsorbed carcinogens (e.g., phthalates, bisphenol A, heavy metals) further amplifies this risk via the so-called “Trojan horse” mechanism [135,139].

Thus, while causality remains to be fully established in humans, there exists strong biological plausibility and converging preclinical data suggesting that MNPs originating from the oral cavity—particularly those released from dental practices and materials—may serve as initiators or promoters of malignancy across gastrointestinal, hepatic, and reproductive systems. Given the common presence of polymer-based dental materials in routine care, this pathway merits investigation and potential regulatory reconsideration.

Table 3 synthesizes the main findings concerning the potential relations between MNPs and oral health status, systemic toxicity, and cancerogenesis.

### 3.5. Dental Practice/Materials

#### 3.5.1. Professional-Use Dental Materials

In the dental setting, the professional use and handling process of polymer-based materials, employed widely in restorative, prosthetic, oral surgery, and orthodontic therapies, may represent a relevant source of MNPs release. Indeed, due to their properties, these materials are susceptible to fragmentation and hydrolytic or thermal degradation processes [140], which may occur both during clinical procedures and intraoral exposure, potentially contributing to occupational and patient-related MNPs exposure.

##### Resin-Based Composites

Among restorative dental materials, resin-based composites have emerged as a source of MNPs release within clinical settings, due to their complex organic–inorganic structure that combines unreacted dimethacrylate monomers, including Bisphenol A-glycidyl methacrylate (Bis-GMA), Urethane dimethacrylate (UDMA), and Triethylene glycol dimethacrylate (TEGDMA) with nanosized silica or zirconia-based fillers [141]. While these materials are widely used in conservative and restorative dentistry owing to their mechanical and aesthetic properties, their nature may cause degradation and the release of micro- and nano-particles, exposing both the patients and the dental personnel to the risk of exposure.

In particular, it has been demonstrated that during clinical procedures such as finishing, polishing, or removal of restorations, clouds of composite-derived aerosol are consistently generated, with median particle diameters between 38 and 70 nm and concentrations often exceeding 10^6^ particles/cm^3^ in the breathing zone of the dental practitioner [141]. Furthermore, even under ideal conditions, the degree of monomer conversion in composite resins rarely exceeds 75%, and may drop below 40% in the deeper strata of the restoration or in the oxygen-inhibited superficial layer, leaving behind a biologically active fraction of unpolymerized monomer that may be progressively released through enzymatic degradation or mechanical wear [141], thereby potentially exposing not only dental professionals to a potential inhalation risk during clinical handling, but also patients to both inhalation and ingestion routes of exposure throughout the functional lifespan of the restoration.

Supporting this concept, in vitro studies have demonstrated that exposure to composite-derived MPs may elicit innate immune activation. Indeed, experiments on RAW264.7 macrophages have shown increased production of reactive oxygen and nitrogen species, along with upregulation of IL-6 and TNF-α, particularly when exposed to particles under 5 μm, which are more readily internalized and metabolically disruptive [142,143,144,145].

Although polymerized monomers exhibit lower reactivity than their free or partially cured counterparts, higher concentrations of composite extracts still led to elevated expression of TNF-α and IL-1β, coupled with reduced levels of regulatory cytokines such as IL-10 and TGF-β, suggesting a shift toward a sustained pro-inflammatory state with potential tissue implications [142,143,144,145].

Notably, the role of high-speed rotary instrumentation in generating aerosolized MPs is particularly relevant, since it promotes not only the liberation of nanoscale fillers and resin debris, but also their spatial dispersion across the operatory setting, often beyond the immediate procedural field [141,142]. While high-volume evacuation systems can mitigate but not fully eliminate airborne exposure, evidence suggests that a considerable fraction of these particles remain suspended long enough to be inhaled or to deposit on mucosal surfaces and clinical equipment [142]. Indeed, it has been reported that dental hospitals exhibited a markedly higher daily MP deposition (1083.80 ± 133.7 MPs/g/day) compared to private clinics (587.60 ± 184.9 MPs/g/day), with particle abundance increasing by 31% during the winter months, likely reflecting reduced ventilation rates [142]. Polyethylene terephthalate was the most abundant polymer identified (39%), and based on average exposure metrics, the estimated inhalation intake of MPs for dental professionals reached 29.45 MPs/g/day (7659.12 MPs/g/year) in hospital settings, compared to 20.23 MPs/g/day (5259.85 MPs/g/year) in private dental clinical environments [142]. This discrepancy may plausibly reflect a combination of several factors, including longer operating durations, higher staff density, crowded training activities, and frequent use of polymer-rich consumables in hospital settings. Additionally, suboptimal ventilation or overcrowding in hospital rooms could increase aerosol accumulation, contributing to a higher airborne MP burden. Notably, it was observed that female dental professionals exhibited 1.1 times greater inhalation exposure than their male counterparts [142]. It might be likely that such gender-related difference is potentially attributable to subtle physiological differences, including women’s slightly higher baseline respiratory rate, smaller airway calibers, and potentially greater particle deposition efficiency within the respiratory tract, particularly for ultrafine particles [146,147]. These factors, combined with occupational dynamics and ergonomics such as proximity to the aerosol source, task distribution, or differences in personal protective equipment fit, may contribute to the observed gender-related disparity in microplastic exposure among dental professionals.

Although the acute cytotoxicity of these exposures may appear low, it might be conceivable that repeated inhalation of composite-derived MPs and NPs, especially those capable of deep alveolar penetration, could predispose dental practitioners to a range of respiratory affections, from chronic bronchial irritation to long-term oxidative and inflammatory damage, particularly when compounded by additional exposures such as degraded glove particles or plasticizers shed from personal protective equipment and barrier materials. In this context, the hypothesis of an “additive risk”, arising from professional simultaneous inhalational and dermal contact with various plastic-derived particulates is plausible, considering that co-exposure may intensify epithelial reactivity, amplify cytokine release, or promote synergistic redox stress, especially when skin microabrasions or mucosal breaches are present. Supporting this, several studies suggest that dental care workers exhibit radiographic lung changes consistent with chronic particulate exposure, and there is evidence indicating an elevated lung cancer mortality rate among workers involved in plastic and resin research compared to the general population [142,148,149]. Moreover, microplastic exposure has been implicated in exacerbating allergic asthma and fostering interstitial lung diseases [142,150].

Conversely, for patients, the exposure profile shifts toward chronic ingestion, especially in the case of composite restorations and removable prostheses that remain in continuous contact with saliva. While environmental ingestion of MPs through water and food has been widely acknowledged [35,36], it might be conceivable that the cumulative burden in dental patients is significantly amplified by the intraoral presence of restorative materials, which may persist for five or more years before undergoing further degradation [141,151,152]. This prolonged mucosal interface may act as a sustained source of polymeric debris and submicron particles, some of which are likely to be swallowed and enter the gastrointestinal tract [153,154]. Though most particles above 20 μm are probably excreted, smaller MPs and particularly NPs under 5 μm might cross the gut epithelium, disrupt tight junctions, or interact with resident immune cells, potentially contributing to altered gut homeostasis. The toxicological relevance of such exposure is further underscored by recent in vitro studies showing that MPs derived from composite or denture resins can induce ROS production, mitochondrial dysfunction, and upregulation of inflammatory mediators such as IL-1β and TNF-α in keratinocytes and macrophages [140,142].

Furthermore, beyond their direct toxicological and immunological implications, the systemic implications of chronic exposure to MNPs and residual monomers released from dental composites may also need attention. These particles, present in direct and indirect restorations as well as in pit-and-fissure sealants used in both pediatric and adult populations, may leach into the oral cavity over time. Several of the released components, such as Bis-GMA and other bisphenol-A-derived analogues, have shown endocrine-altering potential, by interfering with hormonal signaling pathways regulating growth, metabolism, and immune function [155,156], which might be particularly relevant in developmental-maturation subjects. Thus, the overall effect of intraoral exposure, added to concurrent environmental, dietary, and medical sources of MPs and NPs intake, might contribute to a potentially underrecognized risk profile among dental patients.

It is conceivable that respiratory sequelae might be more plausible in dental professionals due to repeated aerosol inhalation, and that dental patients, through intermittent, chronic low-level ingestion, could experience adverse gastrointestinal effects, including mucosal inflammation, dysbiosis, or immunomodulation. Furthermore, considering that residual monomers and degradation byproducts may act as haptens, it might be plausible to hypothesize allergic manifestations, genotoxic alterations, or endocrine-disruptive pathways, especially in susceptible individuals, which may highlight the need of a more sustainable dental practice which should take into account not only ecological risk, but also the inhalation, contact, and ingestion dynamics, as well as their cumulative effects across different populations and exposure routes.

##### Clear Aligners

Clear aligners (CAs) have gained widespread use in modern orthodontics due to their aesthetic properties, removability, patient comfort, and digital work-flow customization [157,158].

Most aligners are made of thermoplastic polymers, including polyethylene terephthalate glycol-modified (PET-G), polyurethane (PU), or polypropylene-based components [157,159]. These materials, while mechanically robust and optically clear, might be susceptible to physicochemical degradation under certain intraoral conditions. Indeed, continuous exposure to salivary enzymes, cyclic mastication, bruxism, acidic beverages, thermal fluctuations, and hygiene practices may induce hydrolytic chain scission, surface abrasion, or component ageing and oxidative wear [160], causing a potential structural integrity impairment over time, as well as the progressive release of MNPs, which could lead to oral and systemic implications.

A recent systematic review [161] highlighted the release of MPs from commercially available CAs in simulated intraoral aging. Among the studies included, one in vitro investigation [162] reported MP release ranging from 1124 particles per sample for PET-G-based aligners, to 5768 particles for PU-based devices.

PU-containing aligners released a higher number of MPs compared to PET-G alternatives, potentially highlighting the CAs’ composition and mechanical properties in degradation processes. Most MPs measured between 5 and 20 μm in size, whereas smaller particles (<5 μm) were identified less frequently, suggesting variability in particle size distribution across brands [162].

Eliades et al. [163] examined NPs release under harsher simulated aging conditions involving acidic pH, thermal cycling, and simulated mastication. The analysis showed NP concentrations ranging from 1.2 × 10^6^ to 4.8 × 10^6^ NPs/mL in artificial saliva, with particle sizes between 75 and 120 nm and zeta potential values of −19.4 to −23.7 mV, suggesting a potential colloidal stability and bioadhesive interactions. Surface topography analysis confirmed a relevant increase in roughness and microcrack formation following aging, potentially facilitating both particle detachment as well as biofilm colonization [163].

The current evidence remains debated regarding the biological effects of aligner materials. Some in vitro studies have reported a potential cytotoxic impact in certain thermoformed polymers [164]. For instance, the exposure of human gingival fibroblasts to several aligner materials for 14 days showed a reduction in cell viability, with a citotoxicity ranging from 65% to 85% among different CAs formulations. Notably, the thermoforming process appeared to influence cytotoxicity in specific materials, suggesting that fabrication methods may influence the biological reactivity of these polymers [164].

In addition, release from some clear aligners has also been shown to impair viability and barrier function in oral epithelial cells [165]. Indeed, cells exposed to some clear aligner material eluates showed a decrease in optical density, disrupted membrane integrity, and compromised epithelial micromotion, especially under mechanical stress [165]. Such alterations could potentially facilitate inflammatory or immune responses within the oral environment.

Although most available data derive from in vitro investigations, clinical evidence regarding the biological effects of MNPs from clear aligners remains limited and partially inconclusive. A recent systematic review [166], evaluating both experimental and clinical studies, reported that while aligners are generally well tolerated, mild cytotoxicity and localized mucosal irritation have been occasionally observed. These effects depended on the polymer type, such as PU or PET-G, the thermoforming process, and intraoral factors including temperature, pH, and mechanical stress [166]. Notably, PET-G-based devices such as Invisalign reported a lower inflammatory profile compared to some PU-based counterparts, supporting the likely more favorable biocompatibility in vivo [166]. Nevertheless, individual variability, oral hygiene, salivary properties, and treatment duration likely influence the extent and clinical relevance of these interactions [167].

Notably, there is increasing evidence of the potential for immunological reactions to aligner or plastic bracket-derived polymers [168]. For instance, CAs and esthetic brackets made of PU or polycarbonate may elicit hypersensitivity reactions in susceptible individuals [168]. These materials are prone to intraoral degradation due to thermal, enzymatic, and mechanical factors, and may consequently release byproducts such as BPA or hexamethylene diisocyanate (HDI) [169].

Clinical observations have linked these degradation products to allergic manifestations ranging from localized gingival inflammation to perioral manifestations. For instance, a documented case of hypersensitivity to HDI in a patient undergoing clear aligner therapy supports the hypothesis of isocyanate-related allergic responses [168]. These reactions may be induced by the formation of haptens, which may potentially be derived from MNPs release.

The available data are heterogeneous, including in vitro studies suggesting that many aligner materials are safe, while others report a potential cytotoxic or immunogenic effect, particularly under conditions of prolonged exposure or mechanical stress. These differences between in vitro and in vivo studies may derive from variation in polymer composition, patient usage, or study methodologies. Moreover, the non-standardized protocols for assessing MNPs release and limited long-term clinical follow-up represent a significant gap in the current literature. Notably, considering that patients typically use between 20 and 40 pairs of aligners (140/280 days–280/560 days) per treatment cycle, each worn for up to 22 h per day [170,171], chronic low-level exposure to MNPs may potentially disrupt epithelial homeostasis, alter immune responses, and affect the oral microbiota.

##### Dental Prosthesis and Impression Materials

The extensive use of polymeric materials in prosthodontics and dental impression techniques in modern dental care is increasingly associated with environmental and health-related concerns due to the generation of MNPs [142]. Among these materials, polymethyl methacrylate (PMMA) is the most common fabrication material of denture bases, temporary crowns, and prostheses [142]. Moreover, polyethylene terephthalate (PET) is also frequently used for the fabrication of dental prosthetic components, such as retention capsules used in implant-supported overdentures [172,173].

However, handling processes including trimming, grinding, and polishing of PMMA-based prosthetic elements could lead to material fragmentation in MNPs, whose size and morphology vary in relation to the finishing techniques employed and the material aging or degradation [174]. Clinical and laboratory dental practices involving high-speed rotary instrumentation are particularly implicated in the generation of airborne plastic particles within the respirable size range (<10 µm), which may accumulate in dental treatment areas, infiltrate suction systems, and bypass filter systems. These particles, if aerosolized, can remain suspended in the environment, representing a plausible inhalation risk for dental personnel and technicians, especially in inadequately ventilated settings or workplaces not providing proper particulate filtration systems [142]. In addition to that, one study [142] evaluated the potential impact of denture-derived MNPs exposure on patients by dental prosthetic devices immersion in artificial saliva for seven days under different cleaning protocols, including toothbrushing and cleansing tablets. This in vitro model, designed to simulate real clinical scenarios, showed the release of 200–400 total particles per 1000 mL, with 2–5% identified as MPs. Among the most frequently detected polymers were PET, PMMA, acrylate copolymers, and polystyrene, materials commonly used in dentures and dental crowns [142]. These findings may suggest that even routine maintenance of removable prostheses may contribute to continuous, low-level exposure to MPs in the oral environment.

The release of MNPs extends to impression-taking materials as well. Traditional alginate impressions are biodegradable, but many newer alternatives commonly used, including polyvinyl siloxanes and polymer-based formulations, contain synthetic particles that do not degrade easily and might be fragmented [174]. Whether inadequately rinsed, disinfected, or disposed of, these fragments can enter wastewater systems or remain on reusable trays, acting as silent contributors to MNP release [174]. Routine cleaning and clinical handling may also induce micro-fracturing or abrasion of these materials, promoting further particle dispersion.

Moreover, the increasing reliance on digital workflows in dentistry may also represent a concern. Technologies such as 3D printing and CAD/CAM milling have revolutionized modern prosthodontics precision and customization, and are routinely used for producing surgical guides, temporary and definitive restorations, and dentures [175,176,177]. However, these innovations may cause a relevant environmental cost, since the photopolymer resins employed in stereolithography and digital light processing printers are typically acrylate-based, non-biodegradable, and frequently discarded as uncured remnants, failed prints, or support structures, which can be potentially released into the environment [174]. Simultaneously, manufacturing through CAD/CAM milling of pre-polymerized blocks, such as PMMA or zirconia-reinforced polymers, generates fine particulate debris, which tends to accumulate in the milling chamber or is flushed out with coolant solutions [174]. In the absence of proper filtration methods, these MPs may escape into standard drainage systems, further contributing to dental settings and environmental microplastic loads [142].

In addition to that, the chronic inhalation of MPs and NPs by dental professionals represents a poorly addressed health risk. Particles from dental resins were linked to oxidative stress, inflammation, and cytotoxic effects in human oral keratinocytes and macrophages, suggesting a potential link between prolonged exposure and mucosal/systemic inflammatory responses [142,178]. PMMA-derived MPs were found in human biological compartments, including the bloodstream, liver, and heart, raising concern about potential accumulation and long-term toxicity [178]. Given their small size and high surface reactivity, it is plausible that NPs can translocate through epithelial and endothelial barriers, although the exact biodistribution and toxicokinetics in dental contexts remain to be fully elucidated.

In addition to occupational risks, there are potential implications for patients, who may be chronically exposed to MNPs through direct contact with polymer-based prostheses worn intraorally for extended periods. Dentures, retainers, and aligners fabricated from PMMA and other synthetic resins are in continuous contact with saliva, mucosa, and the oral environment. This prolonged contact might facilitate the release of MNPs by mechanical abrasion, enzymatic degradation, or thermal stress. It might be hypothesized that these particles could over time adhere to mucosal surfaces, be inadvertently ingested, or enter epithelial tissues, potentially eliciting local inflammatory responses or contributing to systemic MNPs accumulation.

#### 3.5.2. Home-Use Dental Materials

The incorporation of plastics in home-use oral healthcare products such as toothpastes, mouthwashes, dental floss, toothbrushes, whitening agents, and interdental brushes has raised considerable concern about the potential release of MNPs into the human oral and systemic environment as well as ecosystem pollution [140]. These particles, added as exfoliating microbeads or unintentionally produced through mechanical movements and material aging, may represent a chronic and underestimated source of MNP exposure [140].

##### Toothpaste, Toothbrushes, Dental Floss, and Mouthwashes

Toothpastes are formulated with remineralizing agents, antibacterials, and occasionally probiotics, but also with MPs introduced to enhance whitening, scrubbing, and polishing efficacy [140,179,180]. These materials, intended to be rinsed, are ultimately discharged into wastewater systems and may contribute to aquatic contamination and bioaccumulation in marine organisms [15,140]. Chemical analyses have identified polyethylene, polypropylene, polyvinyl chloride, polyamide, and cellophane as the main MP content in toothpaste, with polyethylene being the most prevalent, with estimated concentrations of up to 1.8% in some formulations [15,140].

Moreover, the brushing process may further contribute to this contamination. An in vitro simulation showed that brushing with commercial toothbrushes led to measurable MP release into artificial saliva, suggesting that bristle degradation under repetitive and continuous stress may contribute to long-term oral and gastrointestinal exposure [15]. Moreover, toothbrush bristles, commonly made of nylon or thermoplastic elastomers, undergo intraoral aging, microabrasion, and fragmentation, especially involving the gingival margin, by releasing fibers and fragments that can interact with both the oral epithelium and subgingival tissues, further supporting the need and advice for periodical toothbrush substitution. An Indian analysis estimated an annual exposure of 48,910 particles per person via toothbrush use alone [15], potentially highlighting the impact of such particle release on the oral microbiome.

Dental floss is typically manufactured from non-biodegradable, non-recyclable materials such as waxed nylon or polytetrafluoroethylene (Teflon), which are associated with single-use plastic pollution [129]. Additionally, some floss products are coated with per- and polyfluoroalkyl substances (PFASs) to reduce friction and facilitate interproximal passage [129]. These persistent chemicals may leach out during decomposition or be released directly into the oral cavity. Notably, the mechanical action of flossing might result in micro-injuries and gingival bleeding, potentially allowing MPs and NPs released from the floss material to come into direct contact with the gingival crevicular fluid and blood. It might be plausible that this interaction between plastic debris and subgingival tissues could represent an unrecognized chronic route of exposure. The oral microbiome or its byproducts might gain transient or sustained access to the systemic circulation, raising questions about the immunological and inflammatory consequences of repeated MNPs-mediated interactions in the oral epithelial barrier.

Mouthwashes, commonly used as adjuncts to hygiene routines, can also be vectors of MNPs [129]. Some formulations contain suspended MPs to enhance texture characteristics, while active ingredients such as triclosan are frequently included for their antibacterial properties [129]. Although triclosan exhibits low acute toxicity, its use remains only partially regulated, and its persistence in the environment and the related potential endocrine-disrupting effects have raised concerns [129]. Daily exposure via mouth rinsing, coupled with high discharge into wastewater (74 billion particles/day in India), underscores its relevance to both personal and environmental health [15].

**Figure 2:**
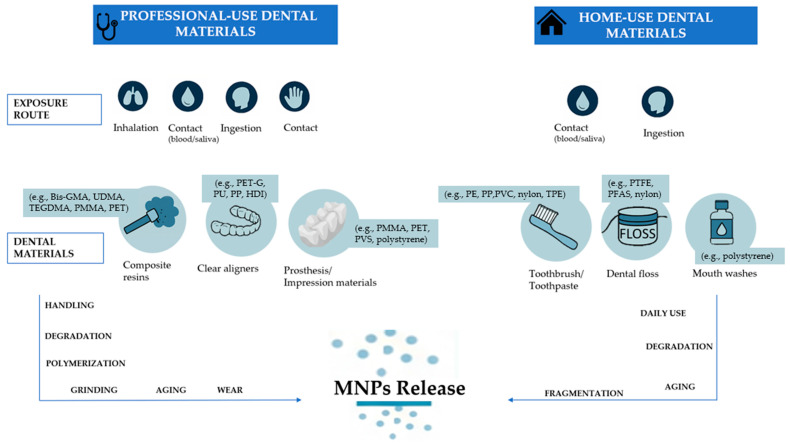
Dental materials (professional and home-use) and the exposure routes involved in the release of MNPs.

It should be considered that not all released MNPs are necessarily ingested or absorbed; many are expectorated. However, partial retention in the oral cavity may allow prolonged contact with mucosal surfaces or incorporation into the oral biofilm. Indeed, in vitro studies have shown that MPs, especially polystyrene-based, can alter fibroblast viability and inflammatory pathways in gingival tissues, by activating cellular signaling and reducing metabolic activity in human gingival fibroblasts [124]. This may support the hypothesis that prolonged intraoral exposure to MNPs may have subclinical or chronic effects on periodontal health. Figure 2 depicts the dental materials (professional and home-use) and the exposure routes involved in MNPs release.

## 4. Discussion

The present narrative review aimed to point out the origin, release mechanisms, physico/chemical properties, and toxicological/biological effects of MNPs from dental sources (e.g., composite resins, clear aligners, orthodontic and prosthetic devices, impression materials), oral care products (e.g., toothpastes, mouth rinses), and environmental exposure.

The presented findings across local and systemic contexts reveal consistent patterns of cellular stress, inflammation, and impaired homeostasis following MNP exposure. While the oral cavity serves as a gateway, the effects reverberate systemically, emphasizing the need for interdisciplinary research, regulatory oversight, and safer material design in dentistry. The biological plausibility of MNP-induced carcinogenesis and systemic disease warrants longitudinal studies to define thresholds of exposure and guide clinical practices. In addition, comparisons with broader systemic findings and speculative links to cancerogenesis and mucosal pathologies are also drawn, particularly in the context of systemic translocation and oral carcinogenic mechanisms [93,115,128].

Locally, MNPs compromise oral mucosal integrity through cytoskeletal disruption, mitochondrial dysfunction, and oxidative stress in gingival fibroblasts [113,124]. These effects mirror systemic epithelial compromise observed in gastrointestinal tissues following chronic oral exposure, with evidence of epithelial thinning and goblet cell loss [65,68,154].

The shared mechanisms—mitochondrial depolarization, ROS production, and impaired repair—suggest that the oral cavity may serve as a sentinel site for systemic epithelial injury. Given the continuous exposure of oral mucosa to both dental-derived and environmental MNPs, it is plausible that early cytotoxic changes seen orally are predictive of downstream systemic tissue compromise.

Dysbiosis of the oral microbiota has not yet been as clearly delineated as in the gut, but emerging parallels are compelling. In murine models, MNPs significantly disrupt gut microbial composition, favoring pro-inflammatory taxa and reducing commensal bacteria, resulting in chronic inflammation and immune dysregulation [59,68,136,153]. Given the anatomical and functional similarities between the gut and oral mucosa-associated microbiota, it is reasonable to speculate that oral colonization patterns may similarly shift in response to chronic MNP exposure. This would explain observed increases in gingival inflammation and could contribute to mucosal degradation and subsequent systemic inflammatory priming [115,132].

Epithelial interaction and intracellular penetration of NPs are pivotal in initiating pro-oncogenic signaling. Correspondingly, salivary proteins such as mucins and low-molecular-weight peptides have been shown to bind to MNPs, forming a bio-corona that facilitates mucosal adhesion and internalization [24,181]. This interaction likely enhances epithelial uptake and prolongs exposure duration, thereby increasing genotoxic potential. This is consistent with findings that internalized MNPs localize to lysosomes and mitochondria, inducing ROS overproduction and cell stress [83,119]. These mechanisms reinforce the hypothesis that variability in salivary composition may influence MNP toxicity profiles.

Experimental exposure to polystyrene NPs in gingival fibroblasts leads to mitochondrial depolarization, ROS overproduction, and genotoxic stress—hallmarks of malignant transformation [83,119]. These mechanisms are reflected in animal studies documenting epithelial thinning and mucosal barrier loss in MNP-exposed gastrointestinal tissues [68,136,153]. The chronicity of exposure and accumulation of DNA damage are consistent with known models of multistep carcinogenesis. Although direct evidence of oral malignancy remains preliminary, histopathological findings align with preneoplastic transformation, emphasizing the carcinogenic potential of MNPs when exposure is sustained.

MNPs originating from professional and home-use dental material can be ingested or inhaled and subsequently translocate to systemic sites, including the liver, spleen, and reproductive tissues [73,128]. Mechanistically, this parallels the findings in oral tissues: mitochondrial dysfunction, oxidative stress, cytokine upregulation, and epithelial barrier breakdown [73]. The systemic consequences—hepatic inflammation, dyslipidemia, immune dysregulation—are reflective of the oral cavity’s role as a gateway for particle translocation. Moreover, endocrine-disrupting chemicals in dental plastics (e.g., BPA, phthalates) are implicated in reproductive toxicity and hormonal imbalance [15,126].

The key oncogenic mechanisms associated with MNPs include suppression of tumor suppressor genes, chronic inflammation, mitochondrial dysfunction, and oxidative DNA damage. These findings are corroborated by systemic models of colorectal and liver carcinogenesis, where chronic MNP exposure activated pro-inflammatory transcription factors such as NF-κB, increased IL-6 and TNF-α expression, and induced genomic instability [90]. Additionally, MPs have been recovered from human para-tumoral tissues in colorectal and prostate cancers [14,102,109], reinforcing their ability to persist and accumulate at sites vulnerable to malignant transformation. The “Trojan horse” phenomenon, whereby MNPs serve as carriers for toxic co-contaminants, adds another layer of risk by facilitating the delivery of carcinogens such as heavy metals and endocrine disruptors [135,139].

### 4.1. Policy and Recommendations for Dental Practice and Related Fields

#### 4.1.1. System-Level Policy Recommendations

In light of the growing body of evidence linking plastics to adverse health and environmental outcomes, the Minderoo-Monaco Commission recommends a coordinated policy response aimed at mitigating exposure, protecting vulnerable populations, and transitioning toward a circular, non-toxic plastics economy [11].

Key priorities and related strategies identified by the Commission include:

- MNPs-free materials: there is a need to establish a global cap on the production of virgin plastics, particularly those derived from fossil fuels [11]. Curbing upstream plastic production is viewed as essential not only to reduce environmental plastic burdens but also to minimize chemical exposures associated with extraction, polymerization, and manufacturing processes [11].
- Chemical compatibility and transparency: there is a necessity of eliminating hazardous substances from all stages of the plastic life cycle. This includes the targeted removal of persistent, bioaccumulative, and toxic chemicals, endocrine-disrupting chemicals, and other substances of very high concern [11]. Such a transition requires the development and enforcement of stringent regulatory frameworks that mandate full transparency and disclosure of all chemical constituents used in plastic materials [11]. Chemical transparency is considered a precondition for effective risk assessment, safer substitution, and innovation in green chemistry [11].
- Adoption of health-protective principles into United Nation Global Plastics Treaty: the integration of health-protective principles into the United Nations Global Plastics Treaty is currently under negotiation [11]. The treaty should prioritize public health as a core objective, impose binding obligations on countries to reduce harmful plastic production and use, and include mechanisms for supply chain accountability. Additionally, it should require the systematic monitoring and public reporting of plastic-related human exposures and health effects [11].
- Scientific research and economic reform: there is a need for sustained investment in scientific research [11]. This includes biomonitoring initiatives to track internal human exposure to micro- and NPs, longitudinal epidemiological studies to assess long-term health outcomes, and research in green chemistry to develop safe and sustainable alternatives to conventional plastics [11]. There is a need for public engagement and economic reform. Public awareness campaigns should be mobilized to inform consumers and stimulate demand for safer materials. Simultaneously, economic tools such as plastic taxation, extended producer responsibility schemes, and financial incentives for sustainable innovation should be deployed to reflect the true environmental and health costs of plastics and to drive systemic change [11].

#### 4.1.2. Clinical Implications, Actionable Recommendations, and Operative Measures in Dental Practice

**Figure 3:**
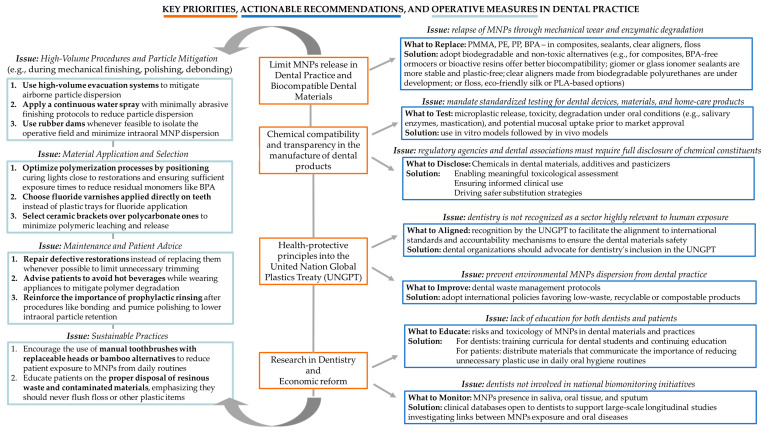
Frameworks on the key priorities in dental practice according to the global policy priorities recommended by the Minderoo-Monaco Commission, related actionable recommendations in dental settings (identification of major issue, focus, and solution), and operative measures in dental practice [5,11,15,24,120,129,140,162,181].

Given the emerging evidence linking MNPs from dental materials and oral-care products to adverse local and systemic health effects, targeted actions in dentistry are warranted. The following key priorities, actionable recommendations, and operative measures in dental practice align with global policy priorities and are tailored to the unique role dental practice plays in MNP exposure and mitigation [11] (Figure 3).

### 4.2. Limitations

Given the narrative design of the present narrative review, a key limitation is its susceptibility to potential bias. Unlike systematic reviews, the narrative review methodology does not employ a pre-defined and rigorous quality appraisal. This approach can lead to the inclusion of studies with high risk bias, precluding the possibility of conducting an unbiased synthesis of the available data. Furthermore, the absence of a quantitative meta-analysis prevents a statistical assessment of the evidence’s strength.

In addition to that, the literature search, while comprehensive, may have inadvertently missed relevant studies, particularly those published in languages other than English or in non-indexed sources. While these factors limit the review’s capacity to serve as a definitive guideline, it remains a valuable tool for summarizing the current state of knowledge and identifying key areas for future research.

Moreover, the available evidence is largely derived from animal models or in vitro experiments, which may not fully replicate human physiology or clinical contexts, while human studies had short-term follow-ups. As a result, the ability to establish causal relationships remains restricted.

### 4.3. Future Research

Future research should prioritize longitudinal epidemiological studies to clarify the causal relationship between chronic oral exposure to MNPs and the development of oral diseases and malignancies. While in vitro and in vivo models suggest cytotoxic, genotoxic, and pro-inflammatory mechanisms leading to carcinogenesis, confirmation in human populations remains limited [55,88,145,153,178].

The characterization of MNP interactions with the oral microbiome and salivary proteome requires expansion. Proteomic and metagenomic studies should assess how MNPs alter microbial composition and immune signaling, potentially fostering dysbiosis and chronic inflammation [15,140]. Moreover, the impact of bio-coronas formed in the oral cavity on nanoplastic uptake and toxicity remains largely unexplored.

Regulatory frameworks must evolve to account for the environmental and health risks posed by plastic-containing dental materials and hygiene products. Despite the detection of MNPs in saliva, sputum, and dental materials—even among healthy individuals with no occupational exposure—most commercial products remain unregulated in terms of microplastic content [15,140].

Additionally, innovations in dental material science are needed to develop biodegradable, non-toxic alternatives to current polymeric composites, sealants, and flosses. Sustainable manufacturing and disposal protocols for oral healthcare products should be integrated into broader environmental health strategies [15,140].

Ultimately, interdisciplinary research should investigate the potential therapeutic applications of engineered nanoparticles derived from plastic analogs in drug delivery and cancer treatment—an emerging paradox that may significantly impact our understanding of plastic biocompatibility and toxicity.

## 5. Conclusions

This narrative review explored the current evidence regarding the sources, release mechanisms, physicochemical properties, and toxicological or biological effects of micro- and nanoplastics (MNPs) released from dental materials and oral care products, with a particular focus on their combined impact with environmental exposure and their implications for oral and systemic health.

The present findings highlighted that several materials and procedures routinely used in dental practice, including resin-based composites, clear aligners, denture bases, impression materials, and finishing or polishing techniques, may release MNPs into the oral environment. Similarly, home-use products like toothpastes containing polyethylene-based abrasives, toothbrush bristles made of nylon, and plastic-coated dental floss may act as additional sources of MNPs, contributing to daily exposure through ingestion or mucosal contact.

Evidence suggests that these particles can cross biological barriers, persist in tissues, and induce a variety of cellular responses, including oxidative stress, mitochondrial dysfunction, inflammation, and DNA damage, with potential implications for long-term health and carcinogenic risk.

Although direct clinical evidence remains limited, the biological plausibility of these effects—together with the detection of MNPs in saliva, sputum, gastrointestinal tissues, and even para-tumoral specimens—raises questions about the cumulative burden of chronic exposure, particularly through the oral route. The potential involvement of MNPs in altering the oral microbiota, damaging mucosal barriers, and acting as carriers of endocrine-disrupting substances reinforces the need to further explore this topic extensively.

In light of these findings, promoting a more conscious approach to material selection and handling in dental settings might be beneficial. While further studies are needed to better define thresholds of biological relevance, it may be prudent to encourage the development and testing of alternative biomaterials. Additionally, increasing awareness among clinicians, manufacturers, and patients regarding the possible release of MNPs from commonly used materials—both in professional and at-home care—could support more informed decision-making and gradual improvements in clinical protocols.

## Figures and Tables

**Table 1:** Summary of exposure and transmission pathways of MPs and NPs, including primary environmental sources, affected physiological systems, and their corresponding adverse health effects.

Exposure Route	Primary Sources	Target Systems	Health Effects	Description	Common Sources	Potential Health Impact
Ingestion [4,7,14,20,34,35,36,37,38,39,40,41,42,43,44,45,46,47,59,65]	Contaminated food and water, seafood, salt, tea, dairy	Gastrointestinal tract, liver, kidneys	Gut barrier disruption, inflammation, microbial dysbiosis, tissue accumulation	Entry via consumption of contaminated food, water, seafood, salt, tea, dairy, and bottled water	Seafood, drinking water, table salt, tea bags, dairy, bottled water	Intestinal inflammation, disruption of microbiota, systemic distribution via M cells
Inhalation [2,48,49,50,51,52,53,57,58]	Airborne particles from textiles, industrial emissions, and sea spray	Respiratory tract, brain	Pulmonary inflammation, oxidative stress, DNA damage, potential neurotoxicity	Entry via breathing airborne MPs/NPs from textiles, industrial emissions, indoor dust, and sea spray	Synthetic textiles, industrial processes, sea breeze, atmospheric transport	Respiratory tract irritation, inflammation, potential neurotoxicity via blood–brain barrier
Dermal Contact [26,27,54,55]	Cosmetics, personal care products, airborne fallout	Skin, immune system	Allergic reactions, oxidative stress in epithelial cells, and potential for deeper tissue exposure with NPs	Entry via skin contact with cosmetic products or environmental fallout; more plausible with NPs	Cosmetics (e.g., exfoliants, face washes), personal care products, airborne particles	Skin irritation, immune activation, potential oxidative stress in epithelial cells

Abbreviations: microplastics, “MPs”; nanoplastics, “NPs”; deoxyribonucleic acid, “DNA”; microfold cells, “M cells”.

**Table 2:** Potential carcinogenic mechanisms of micro- and NPs (MNPs).

Mechanism	Description
Accumulation and Bioavailability	MNPs can enter the body through ingestion, inhalation, and dermal exposure. Once internalized, they are distributed to organs such as the liver, kidneys, spleen, lungs, and reproductive tissues. Their bioavailability is influenced by size, surface chemistry, and the ‘Trojan horse’ effect—co-transport of pollutants [22,23,66,67,68,69,70].
Cellular and Molecular Toxicity	MNPs cause mitochondrial dysfunction, oxidative phosphorylation impairment, lysosomal permeabilization, ER stress, and apoptosis, contributing to tumor aggressiveness and therapy resistance [14,88,90,96]. They disrupt autophagy and induce necroptosis and pyroptosis, affecting stem and proliferative cells the most [81,88,90,110,111,112,113].
Microbiota Dysbiosis	MNP exposure alters gut microbiota composition, reducing commensals and promoting pro-inflammatory bacteria. It impairs the gut barrier and stem cell regulation, enhances genotoxin bioavailability, and affects immune and metabolic pathways linked to cancer [72,73,74,75,77,78].
Oxidative and Inflammatory Tumor Microenvironment	MNPs induce oxidative stress and chronic inflammation, leading to ROS overproduction and activation of cytokines (IL-1β, TNF-α, TGF-β) and inflammasomes (NLRP3). These microenvironments promote DNA damage immunosuppressive cell recruitment, the activation of pro-oncogenic pathways (NF-κB, HIF1α), and remodeling of the TME, EMT, angiogenesis, and tumor immune evasion [77,80,83,85,86,114,115,116,117].
Genotoxicity and Epigenetic Alterations	MNPs cause DNA strand breaks, chromosomal instability, and micronuclei. They modulate epigenetic marks like DNA methylation, histone acetylation, and miRNA expression, affecting cancer-related genes and DNA repair [88,89,93,94].
Synergistic Effects with Environmental Contaminants	MNPs adsorb and co-deliver heavy metals, POPs, EDCs, enhancing their bioavailability and toxicity. Co-exposure disrupts endocrine, reproductive, and immune systems, aggravating oxidative and genotoxic effects [71,99,102,110].

Abbreviations: micro- and nanoplastics, “MNPs”; endoplasmic reticulum, “ER”; interleukin, “IL”; tumor necrosis factor, “TNF”; transforming growth factor, “TGF”; tumor microenvironment, “TME”; epithelial–mesenchymal transition, “EMT”; deoxyribonucleic acid, “DNA”; micro-ribonucleic acid, “microRNA”; persistent organic pollutants, “POPs”; endocrine-disrupting chemicals, “EDCs”; reactive oxygen species, “ROS”; nuclear factor, “NF”; hypoxia-inducible factor, “HIF”; DNA damage response, “DDR”.

**Table 3:** Main findings on MNPs from oral sources.

Topic	Main Findings
General Health Hazards	MNPs from oral products/dental materials cause oral mucosal damage, dysbiosis, systemic inflammation, genotoxicity, and potential carcinogenesis [15,129].
Systemic Dissemination	Dental-derived MNPs disseminate systemically, necessitating reassessment of dental material safety [62,129].
Oral Cancer Pathogenesis	Chronic oral exposure to MNPs causes oxidative stress, mitochondrial dysfunction, DNA damage, promoting carcinogenesis [74,119,128].
Real-World Exposure	MNPs (PE, PET, PS) found in human saliva and sputum; persistent contact risks inflammation and dysplasia [24,120].
Oral Health Effects	MNPs reduce gingival cell viability, disrupt redox homeostasis, and impair oral barrier function [124,125].
Oral Microbiota and Inflammation	MNPs alter oral microbiota, trigger cytokine release (IL-1β, IL-6, TNF-α), leading to periodontal damage [128].
Systemic Toxicity	Oral-derived MNPs translocate to organs, induce oxidative stress, immune and endocrine disruption.
Endocrine Disruption	BPA, phthalates from dental plastics mimic hormones, affecting reproductive and thyroid systems [102,133,134].
Trojan Horse Effect	MNPs carry toxicants like metals and antibiotics, enhancing systemic toxicity [135,139].
Cancerogenesis	MNPs promote carcinogenesis via DNA damage, immune modulation, and microbiota dysbiosis [5,62,136].

Abbreviations: micro- and nanoplastics, “MNPs”; polyethylene, “PE”; polyethylene terephthalate, “PET”; polystyrene, “PS”; deoxyribonucleic acid, “DNA”; interleukin, “IL”; tumor necrosis factor, “TNF”; bisphenol A, “BPA”.

## Data Availability

No new data were created or analyzed in this study. Data sharing is not applicable to this article.
